# Supplementary material for: Tyrosine residues initiated photopolymerization in living organisms
Source: Nat Commun. 2023 Jun 16;14:3598. doi: 10.1038/s41467-023-39286-8 (PMC10276049; doi:10.1038/s41467-023-39286-8)
Supplement: Supplementary file 1 — Supplementary Information [file 41467_2023_39286_MOESM1_ESM.pdf]

Supplementary Information

Tyrosine residues initiated photopolymerization in living organisms

Mei Zhu, Shengliang Wang, Zhenhui Li, Junbo Li, Zhijun Xu, Xiaoman Liu and Xin Huang**

MIIT Key Laboratory of Critical Materials Technology for New Energy Conversion and Storage, School of Chemistry and Chemical Engineering
Harbin Institute of Technology
Harbin, 150001, P. R. China
E-mails: liuxiaoman@hit.edu.cn; xinhuang@hit.edu.cn

Chemicals

Bovine serum albumin (BSA, Sigma-Aldrich, >98%, *M*w 66 kDa), Lysozyme (human, Sigma-Aldrich, ≥90%, *M*w 16.5 kDa), Glucose Oxidase (GOx, Sigma-Aldrich, from Aspergillus niger, *M*w 160 kDa), Human Serum Albumin, fraction V (HSA, Shanghai yuanye Bio-Technology, 96-99%, *M*w 66 kDa), Ribonuclease A (RNase-A, biosharp, Mw 13.7 kDa), N-hydroxysuccinimide 2-(dodecylthiocarbonothioylthio) isobutyrate (Sigma-Aldrich, 98%), Fluorescein diacetate (FDA, Aladdin, 97%), Propidium iodide (PI, Aladdin, 94%), N,N-Dimethylacrylamide (DMA, Aladdin, >99.0%, stabilized with MEHQ), Ethylene glycol methyl ether acrylate (MEA, Aladdin, 98%), Methoxypolyethylene glycol amine (PEG-NH_2, Aladdin, *M*w ~2000), N-isopropylacrylamide (NIPAM, Aladdin, 98%, stabilized with MEHQ), Poly(ethylene glycol) methyl ether acrylate (PEGA, Aladdin, 100 ppm BHT and 100 ppm MEHQ as inhibitors, *M*n~480), 4-acryloylmorpholine (AML, Aladdin, >98%, stabilized with MEHQ), 4-vinylbenzenesulfonic acid sodium salt (Nass, Aladdin, 90%), Fluorescein O-methacrylate (FMA, Sigma-Aldrich, 97%), Ammonium sulfate ($(NH_4)_2SO_4$, Aladdin, 99%), Dimethyl sulfoxide (DMSO, Aladdin, >99%), N,N-Dimethylformamide (DMF, Aladdin, ≥99.9%), D-(+)-glucose (Sigma-Aldrich, ≥99.5%), Yeast extract (Oxoid), Tryrtone (Oxoid), Tannic acid (TA, Energy, 98%), L-Cysteine (Cys, Energy, >98%), L-Phenylalanine (Phe, Aladdin, 99%), L-Tyrosine (Tyr, Aladdin, 99%), L-Tryptophan (Trp, Aladdin, 99%). All aqueous solutions were prepared using deionized water. The monomers were removed of inhibitors by passing through a column of Al_2O_3. NIH-3T3 mouse-sourced stem cells sample was performed from ProCell, Shanghai, China.

Characterization methods

^{1}H NMR spectra of monomer/polymer solutions were measured on a Bruker Avance-400 MHz NMR spectrometer using D_2O or DMSO-d6 as the solvent. Gel permeation chromatography (GPC) was performed on a Waters 1515 Isocratic HPLC pump, in an Intertek 81437 column oven operated at 40°C. DMF containing 10mM LiBr was used as the eluent at a flow rate of 1 mL/min. Polystyrene (PS) standard was used to calibrate GPC for molecular weight measurements. Optical images were performed on a Leica DMi8 manual inverted fluorescence microscope at 40× magnifications. Image J software was employed to measure integrated optical density of SDS-PAGE gel and the statistics of yeast cells. Confocal fluorescence images were performed on a Leica SP8 Laser scanning confocal microscope. Fluorescence spectra was performed by a fluorescence spectrophotometer (PerkinElmer, USA, LS 55). UV-vis spectra and turbidity measurements were measured on a PerkinElmer spectrophotometer (Lambda 750S, USA). Scanning electron microscopy (SEM) images of dried samples were obtained with a SU8000 instrument. Dynamic light scattering (DLS) measurements were performed using Malvern Zetasizer Nano-ZSP at 25°C using a scattering angle of 90°. Sodium dodecyl sulfate-polyacrylamide gel electrophoresis (SDS-PAGE) was conducted at 120 V using Tris-MOPS-SDS Buffer as running buffer. The samples were loaded by mixing 10 μL of measured solution (1 mg/mL) with 10 μL of 2× loading buffer, followed by Coomassie Blue staining. Prepare the staining solution containing 0.1% coomassie R-250 in 40% ethanol, 10% acetic acid and a destain solution containing 10% ethanol and 7.5% acetic acid. Fourier transform infrared spectroscopy (FTIR) measurements were performed on PerkinElmer

spectrometer with a $LiTaO_3$ detector (Spectrum Two, USA). Cyclic voltammetric measurements were conducted on a CHI760E eletrochemical working station (Chenhua, Shanghai, China) connected to a three-electrode system by using a gold disk as working electrode with a 4 mm diameter working area, Ag/AgCl as reference electrode, and a platinum wire as counter electrode. FTO as working electrode was used in experiment of photocurrent responses in 0.3 M Na_2SO_4 solution. Electron spin resonance (ESR) measurement was performed using Bruker EMX PLUS. General instrument parameters were as follows: center field, 3500 G, field scan range, 100 G, microwave power, 6.325 mW, sweep time, 30 s, time constant, 0.01 ms, modulation frequency, 100 kHz, modulation amplitude, 1 G.

Synthesis of PEG-CTA (6)

N-hydroxysuccinimide 2-(dodecylthiocarbonothioylthio)isobutyrate (CTA) was dissolved in 2 mL DMSO, and subsequently 2 mL of DMSO containing PEG-NH_2 (200 mg, 0.1 mmol) was added dropwise into the above stirred solution and reacted at room temperature for 24 hours. Then, the reaction solution was diluted with water and dialyzed (dialysis tube ~500 Da MWCO) extensively against Milli-Q water. The solution was purified by using a filter (0.45 μm) to remove any unreacted solid CTA. After freeze-drying, the yellow product **6** was obtained about 71% yield. ^{1}H NMR spectrum of **6** was shown in Supplementary Fig. 5. ^{1}H NMR (400 MHz, Deuterium Oxide): δ 0.92 (3H, Ha), δ 1.31 (16H, Hb), δ 1.43 (2H, Hc), δ 1.75 (6H, Hf), δ 1.71 (2H, Hd), δ 3.33 (2H, He), δ 3.4 (3H, Hg), δ 3.73 (Hh).

Synthesis of the block copolymer 8

First block **7** was obtained by above mentioned method as macromolecular chain transfer agent followed by the addition of the second monomer. Typically, the synthesis of the first block **7**: BSA (20.92 mg, 0.315 μmol), **3** (100 mg, 1.0088 mmol) and **6** (4.75 mg, 2.02 μmol) were dissolved in deionized water (1 mL) in a 5 mL of glass vial sealed with a rubber septa. The reaction mixture was degassed by bubbling argon for 25 minutes. The vial was irradiated for 6 h under violet LED light (20 W, 405 nm) at room temperature. Monomer conversion was 85.7%. Furthermore, by adding the reaction mixture (1mL) into saturated $(NH_4)_2SO_4$ solution (3 mL), proteins and polymers were separated due to salt out. After drying in air, pure **7** was obtained., which was then used as macromolecular chain transfer agents for the synthesis of the block copolymer **8**. BSA (20.92 mg, 0.315 μmol), **3** (100 mg, 1.0088 mmol) and **7** (38 mg, 0.82 μmol) were dissolved in deionized water (1 mL) in a 5 mL of glass vial sealed with a rubber septa. The reaction mixture was degassed by bubbling argon for 25 minutes. The vial was irradiated for 6 h under violet LED light (20 W, 405 nm) at room temperature. Monomer conversion was 80.1%. Monomer conversion was determined by ^{1}H NMR spectrum. The product was dried by freeze-drying and dissolved in DMF for GPC characterization to measure number-average molecular weight (M_n) and the polydispersity (M_w/M_n).

Preparation of polymer nanoparticles by heterogeneous polymerization

The dispersion polymerization of monomer **1** was carried out by **6** as stable block to prepare the nanoparticles. In brief, BSA (16 mg, 0.2407 μmol), **6** (3.84 mg, 1.6368 μmol), **1** (100 mg, 0.7684 mmol) were dissolved in deionized water (1 mL) in a 5 mL glass vial sealed with a rubber

septa. The vial was illuminated under violet LED light (20 W, 405 nm) at room temperature. The process was monitored using UV-vis spectra with turbidity measurements at 650 nm. The nanoparticle size of the various reaction time was determined by DLS. Each reaction aliquot was dried by freeze-drying and dissolved in DMF for GPC characterization to measure number-average molecular weight (Mn) and the polydispersity (Mw/Mn).

Determination of protein concentration by Bradford assay

Bradford assay was employed for quantitative analysis of supernatant protein after BSA-mediated photopolymerization of **1** at different times. Similarly, BSA (16 mg, 0.2407 μmol), **6** (3.84 mg, 1.6368 μmol), **1** (100 mg, 0.7684 mmol) were dissolved in deionized water (1 mL) in a 5 mL glass vial sealed with a rubber septa. The reaction mixture was degassed by bubbling argon for 25 minutes, then irradiated under violet LED light (20 W, 405 nm). The reaction solution was centrifuged after various polymerization times at 13523 × *g* for 5 min and the supernatant solution (5 μL) was diluted with deionized water (495 μL) as the test solution. The test solution (100 μL) was mixed with 1× coomassie brilliant blue G-250 (1 mL) in cuvette and incubated for 3 minutes at room temperature. After that, the absorbance of mixture at 595 nm was monitored to analyze the change of BSA concentration in supernatant.

Experimental procedure for polymerization mediated by the aggregated BSA /denatured BSA

The aggregated BSA aqueous solution was prepared with the saturated $(NH_4)_2SO_4$ aqueous solution as follows: The saturated $(NH_4)_2SO_4$ solution (0.4 mL) and HCl aqueous solution (1 M,10 μL) were added dropwise to BSA aqueous solution (14 mg/mL, 1 mL) to make the BSA solution turbid to obtain the aggregated BSA solution. **6** (5.2 mg, 0.0022 mmol) and **3** (100 mg, 1.0088 mmol) were added to the above aggregated BSA solution and the glass vial was sealed with a rubber septa degassed by bubbling argon for 25 minutes. The vial was illuminated under violet LED light (20 W, 405 nm) at room temperature. After that, the reaction solution was dialyzed (dialysis tube ~35 kDa MWCO) extensively against Milli-Q water to remove salt and unreacted monomer. The solution was dried by freeze-drying and dissolved in DMF for GPC characterization to measure number-average molecular weight (Mn) and the polydispersity (Mw/Mn).

General procedure of ESR experiment

In brief, a glass vial (5 mL) charged with BSA (20 mg, 0.301 μmol), **3** (100 mg, 1.009 mmol), and DMPO aqueous (1 mL, 100 mM) at a molar ratio of [BSA]:[**3**]:[DMPO] = 0.3:1000:100 was sealed with a rubber septa. The reaction mixture was degassed by bubbling argon for 25 minutes. The vial was irradiated under violet LED light (20 W, 405 nm) at room temperature for 3 h, and then the reaction solution was quickly transferred to a capillary tube for ESR measurement.

Synthesis of biomacromolecule chain transfer agent BSA-CTA

BSA-CTA was prepared by utilizing the reaction between the amino groups on BSA and CTA terminal NHS ester. The N-hydroxysuccinimide 2-(dodecylthiocarbonothioylthio) isobutyrate

solution (1 mL of 6 mg/mL DMSO solution) was added dropwise to the BSA solution (25 mL of 4 mg/mL BSA aqueous solution) and stirred at 200 rpm at room temperature for 24 hours. After that, the solution was dialyzed (dialysis tube ~35 kDa MWCO) extensively against Milli-Q water and purified by using a filter (0.45 µm) to remove any unreacted CTA. After freeze-drying, the biomacromolecule chain transfer agent BSA-CTA was obtained.

Synthesis of bioconjugates

BSA-CTA was employed as both an initiator and a chain transfer agent for in-situ polymerization in aqueous solution to synthesize BSA-polymer bioconjugates. Typically, BSA-CTA (20 mg, 0.2949 µmol) and **2** (100 mg, 0.8837 mmol) were dissolved in deionized water (1 mL) in a 5 mL glass vial sealed with a rubber septa. The reaction mixture was degassed by bubbling argon for 25 minutes. The vial was irradiated under violet LED light (20 W, 405 nm) at room temperature. the solution was dialyzed (dialysis tube ~35 kDa MWCO) extensively against Milli-Q water and purified by using a filter (0.45 µm) to remove any unreacted monomer. After freeze-drying, the bioconjugate BSA-poly(**2**) was obtained.

Preparation of Proteinsomes

The proteinosomes were prepared according to the Huang's protocol[1] as follows. The bioconjugate BSA-poly(**2**) synthesized by above method was employed as building block. BSA-poly(**2**) (20 µL, 10 mg/mL, pH 8.0 PBS buffer) contained NHS-PEG_{16}-NHS (0.5 mg, 0.49 µmmol) as cross-linker and sodium carbonate buffer (10 µL, pH 8.0) was mixed with of 2-ethyl-1-hexanol (0.4 mL) followed by shaking the mixture for 10 s. After 12 hours sedimentation, the cross-linked proteinosomes were transfer to deionized water by discarding the upper clear oil layer and washed three time with 1mL 75% ethanol by centrifugation process (1503 × *g*, 4 min). The last time wash instead with deionized water to transfer proteinosomes to aqueous phase.

General procedure of yeast cells modification with CTA

Yeast@CTA cells was prepared by utilizing the reaction between the amino groups on yeast cell surface and CTA terminal NHS ester. Typically, overnight cultured yeast cells were washed three times with water by centrifugation (1503 × *g*, 3 min) and resuspended in deionized water (OD_{600} = 4). N-hydroxysuccinimide 2-(dodecylthiocarbonothioylthio) isobutyrate stock solution (0.2 mL of 5.5 mg/mL DMSO solution) was added dropwise to the yeast cell solution (6 mL, OD_{600} = 4) and stirred at 100 rpm at 4°C for 4 hours. After that, the cells were washed three times with 1 mL water by centrifugation (1503 × *g*, 3 min) and resuspended in deionized water (OD_{600} = 2) to obtain yeast@CTA.

The cell viability test

FDA (5 µL, 4 mg/mL in acetone) and PI (2 µL, 1 mg/mL) were added into the solution with different cell density (1 mL), incubated 20 min at room temperature. After that, the cells were washed three times with deionized water and imaged using a Laser scanning confocal microscope.

General procedure of in-situ modification of polymers on cell surface

Yeast@CTA cells were used as a chain transfer agent. Typically, BSA (10 mg, 0.1505 μmol) and **5** (50 mg, 0.1042 mmol) were added to the yeast@CTA cells solution (1mL, OD_{600} = 2) in a 5 mL glass vial sealed with a rubber septa. The reaction mixture was degassed by bubbling argon for 25 minutes. The vial was irradiated under violet LED light (20 W, 405 nm) for 1.5 hours under shaking at 130 rpm at room temperature. The cells were washed three times with deionized water to obtain yeast cells with polymer modification yeast@poly(**5**).

Cell culture of NIH-3T3 cell

NIH-3T3 cells were cultured in Dulbecco's Modified Eagle Medium (DMEM), containing with 10% (vol/vol) fetal bovine serum and 1% (vol/vol) penicillin/streptomycin at 37°C and 5% CO_2, and then regularly replaced with the fresh medium.

Supplementary Figures

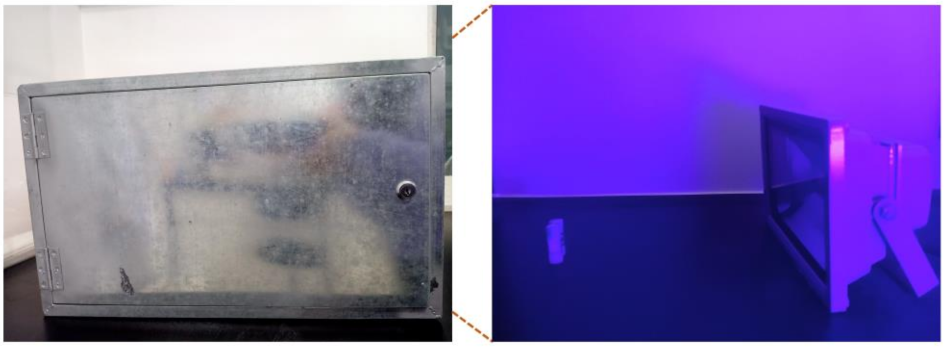

Supplementary Fig. 1. The Photograph of photopolymerization experimental setup.

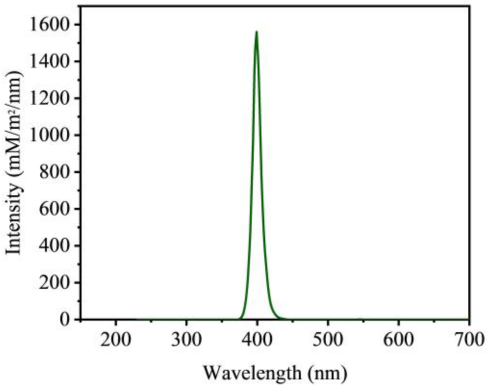

Supplementary Fig. 2. The spectrum of the violet LED light (20 W, 405 nm). The intensity of the UVA of the light is 1.38 mW/cm^2, and all experiments were implemented at the condition.

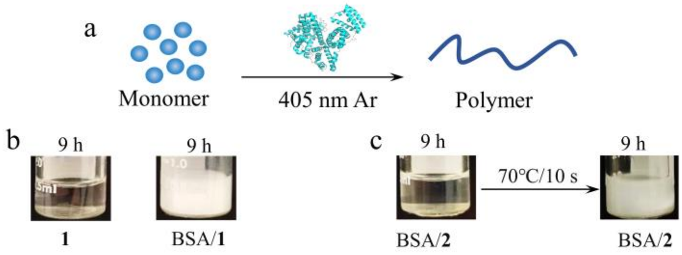

Supplementary Fig. 3. (a) Schematic illustration of BSA-mediated photopolymerization of monomers. Violet LED light (20 W, 405 nm), room temperature, Ar protection. (b) Photograph of **1** and BSA/**1** after 9 hours of polymerization, respectively. The reaction solution containing only **1** still remained transparent, while the BSA/**1** reaction solution produced poly(**1**), a water-insoluble polymer, which made the solution turbid. (c) Photograph of BSA/**2** after 9 hours of polymerization and heated at 70°C for 10 seconds. The white insoluble substance was the temperature-sensitive polymer poly(**2**).

Supplementary Table 1. Polymerization of **1** and **2** at different conditions.[a]

entry	[M]	[BSA]/[M]	Ar	t (h)	light	Conversion
1	**1**	1/2439.34	Y	6	405 nm	>90%
2	**1**	1/2439.34	Y	6	dark	-
3	**1**	1/2439.34	N	6	405 nm	-
4	**1**	0/2439.34	Y	6	405 nm	-
5	**2**	1/2805.41	Y	4	405 nm	>90%
6	**2**	0/2805.41	Y	4	405 nm	-

[a] Polymerization conditions: [BSA]=0.315 mM, [M] = 10% w/v, violet LED light (20 W, 405 nm), room temperature, Ar protection. Conversion ratio was determined by ^1H NMR spectrum in D_2O.

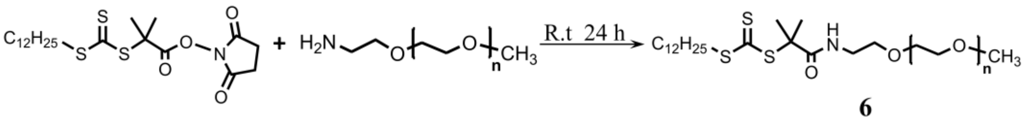

Supplementary Fig. 4. The general procedure of PEG modification of RAFT agent.

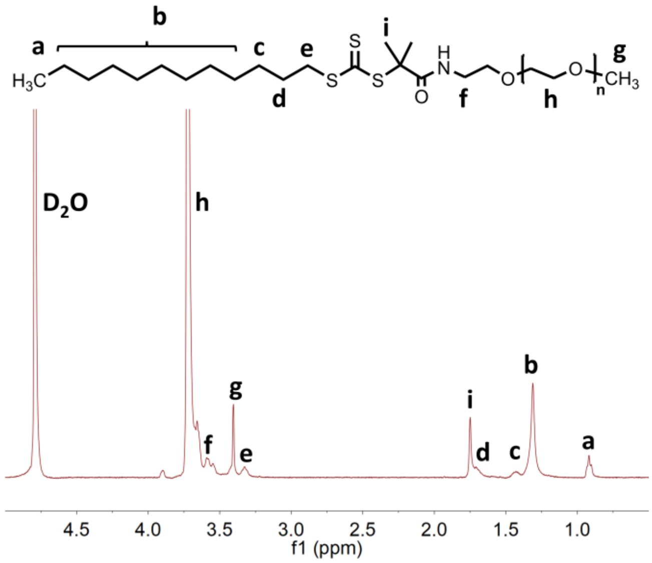

Supplementary Fig. 5. ^1H NMR spectrum of **6** in D_2O.

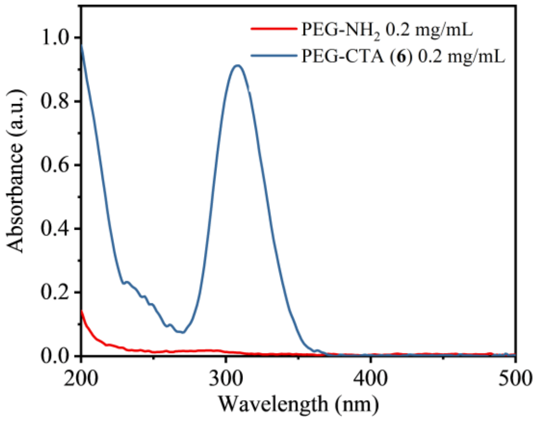

Supplementary Fig. 6. UV-Vis absorption spectrum of $PEG-NH_2$ and PEG-CTA (**6**). The maximum absorbance wavelength of **6** at 310 nm was consistent with the characteristic absorption of CTA, indicating PEG chain was successfully modified by CTA.

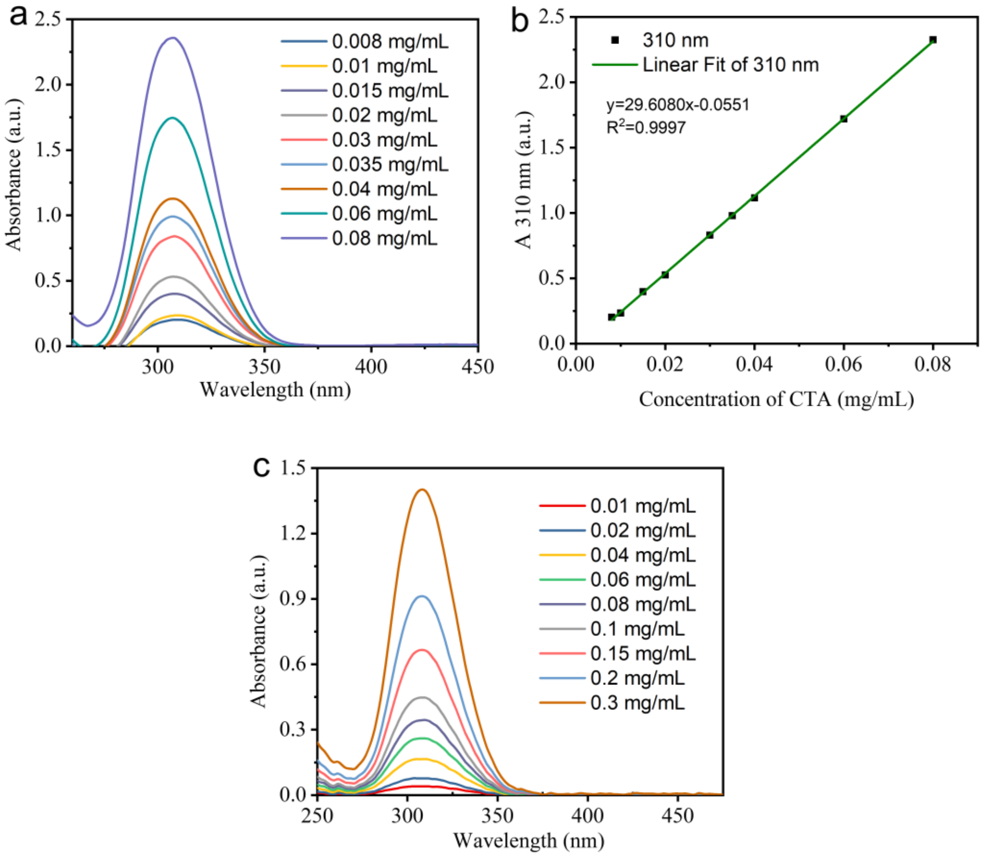

Supplementary Fig. 7. (a) UV-Vis absorption spectrum of a trithiol-RAFT agent under different concentrations from 0.008 to 0.08 mg/mL in DMSO, and (b) corresponding calibration curve based on plotting absorbance at 310 nm against the concentrations of trithiocarbonate. (c) UV-Vis absorption spectrum of the different concentrations of **6**.

Supplementary Table 2. Estimated number of CTA on each PEG, which indicated that almost every PEG chain was modified with CTA.

A310 nm	PEG-CTA (**6**) (g/L)	CTA (μM)	$PEG-NH_2$ (μM)	nCTA:$nPEG-NH_2$
0.03987	0.01	4.02749	4.3	0.93663
0.07694	0.02	7.77214	8.64915	0.8986
0.16508	0.04	16.67566	17.10166	0.97509
0.25974	0.06	26.2378	25.4397	1.03137
0.34434	0.08	34.78372	33.95436	1.02443
0.44677	0.1	45.13075	42.15598	1.07057
0.66292	0.15	66.96528	63.361	1.05688
0.90826	0.2	91.74845	84.05352	1.09155
1.39551	0.3	140.9683	125.4988	1.12326

Supplementary Table 3. RAFT photopolymerization of **3** at the different conditions.[a]

entry	[BSA] mM	[BSA]/[**6**]/[**3**]	light	Conversion
1	0.315	0.125/1/400	405 nm	83%
2	0.315	0.125/0/400	405 nm	92%
3	0	0/1/400	405 nm	-
4	0.315	0.125/1/400	dark	-

[a] Polymerization conditions: [**3**] = 10% w/v, [BSA] = 0 or 0.315 mM, violet LED light (20 W, 405 nm), room temperature, 7 h, Ar protection. Conversion ratio was determined by ^1H NMR spectrum in D_2O.

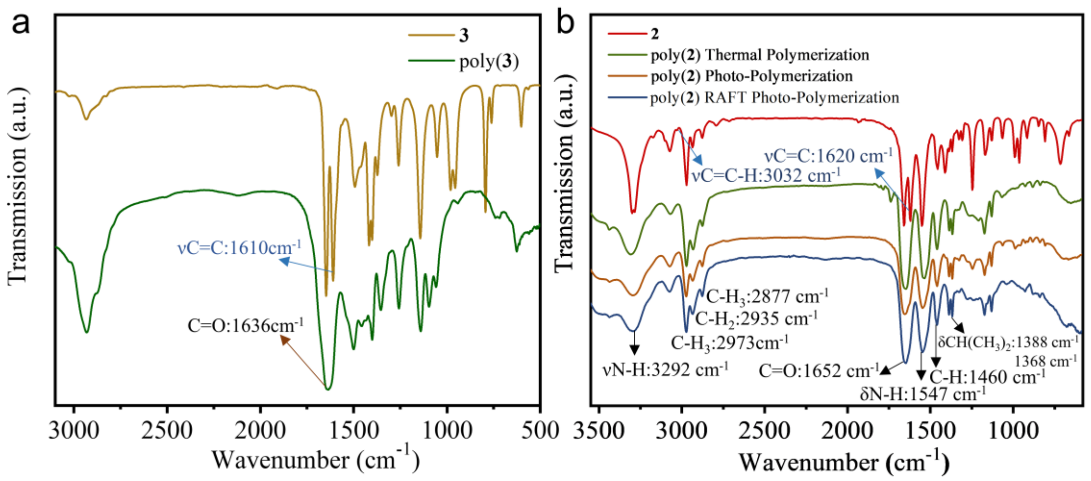

Supplementary Fig. 8. (a) FT-IR spectrum of **3** and poly(**3**) obtained by BSA-mediated RAFT photopolymerization. (b) FT-IR spectrum of **2**, poly(**2**) obtained by RAFT thermal polymerization, poly(**2**) obtained by photopolymerization based on a ruthenium-based photoredox catalyst and poly(**2**) obtained by BSA-mediated RAFT photopolymerization, respectively. The FT-IR spectrum of **3** exhibited the peak at 1610 cm^{-1} which was consistent with C=C stretching vibration. After polymerization, the FT-IR spectrum of poly(**3**) showed the disappearance of the peak, indicating successful polymerization. The FT-IR spectrum of **2** exhibited the peaks at 3032 cm^{-1} (C=C-H stretching vibration) and 1620 cm^{-1} (C=C stretching vibration), while the FT-IR spectrum of poly(**2**) from BSA-mediated RAFT photopolymerization didn't contain the two peaks. The peaks at 1652 cm^{-1} and 1547 cm^{-1} were consistent with the amide I and II band, respectively, and the peak of N-H stretching vibration appeared at 3292 cm^{-1}. While, the peaks of the stretching vibration of C-H were presented at 2973 cm^{-1}, 2935 cm^{-1} and 2877 cm^{-1}. The two peaks with similar intensity indicated the presence of isopropyl groups. Importantly, the poly(**2**) obtained by the three methods exhibited similar FT-IR peaks, revealing same structure of the three poly(**2**).

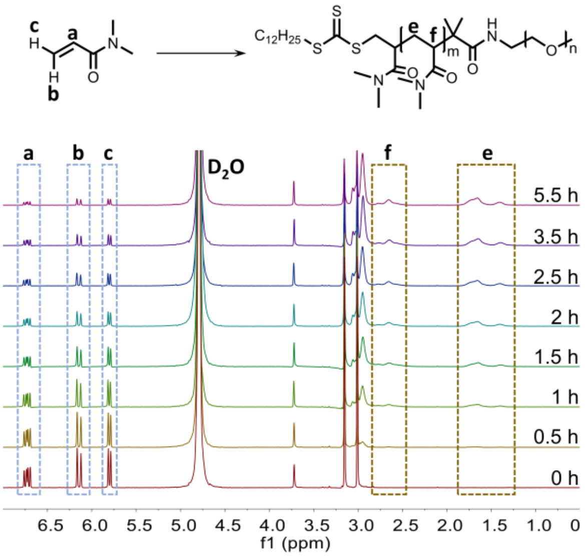

Supplementary Fig. 9. ^{1}H NMR spectrum of BSA-mediated RAFT photopolymerization of **3** at the different time in D_2O. The proton signals of monomer **3** were labeled a, b and c, respectively. With the increase of polymerization time, the proton signals CH_2 and CH of the poly(**3**) main chain appeared gradually, and labeled e and f at δ = 1.22-1.73 ppm and δ = 2.36-2.72 ppm, respectively, accompanied by the decease of the proton signal of monomer. [BSA] = 0.315 mM, [**3**] = 10% w/v, [BSA]:[**6**]:[**3**] = 0.144:1:460, violet LED light (20 W, 405 nm), room temperature, Ar protection.

Supplementary Table 4. RAFT photopolymerization of **1** mediated by the different concentrations of BSA.[a]

entry	[BSA] (mM)	[BSA]/[**6**]/[**1**]	t (h)	M_n (kg/mol)	Mw/Mn	Conversion
1	0.001	5.87×10^{-4}/1/450	6	-	-	-
2	0.005	2.93×10^{-3}/1/450	6	-	-	6%
3	0.025	0.015/1/450	6	108.5	1.34[b]	27%
4	0.1	0.059/1/450	6	122.4	1.19	62%
5	0.3	0.176/1/450	6	136.2	1.15	72%

[a] Polymerization conditions: [**6**] = 1.708 mM, [**1**] = 10% w/v, violet LED light (20 W, 405 nm), room temperature, Ar protection. Molecular weight (*M*n) and polydispersity (*M*w/*M*n) were determined by GPC analysis (DMF used as eluent). Conversion ratio was determined by ^{1}H NMR spectrum in DMSO-*d*6.

[b] When the concentration of BSA was 0.025 mM, the controllability of polymerization was poor with wide molecular weight distribution.

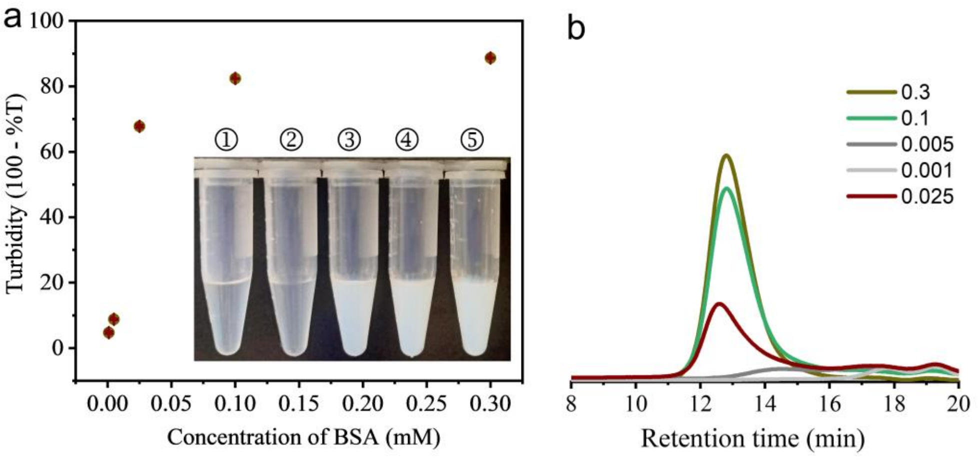

Supplementary Fig. 10. The study of RAFT photopolymerization of **1** mediated by with BSA of different concentrations. (a) The turbidity study characterized by a UV-Vis absorption spectrum at 650 nm. Turbidity was calculated as 100-%T = 100-(100*$10^{-Abs650}$). The inset was the photograph of RAFT photopolymerization of **1** with BSA of different concentrations. (b) The GPC traces of poly(**1**) synthesized with BSA of different concentrations. [**6**] = 1.708 mM, [**1**] = 10% w/v, violet LED light (20 W, 405 nm), room temperature, Ar protection.

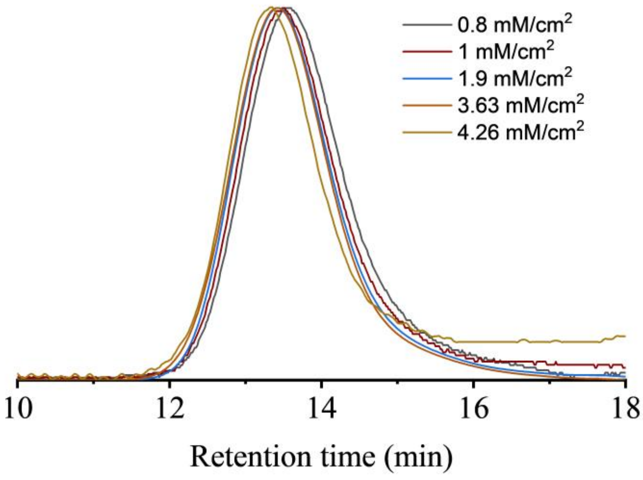

Supplementary Fig. 11. The GPC traces of poly(**3**) synthesized at the different light intensity. [**3**] = 10% w/v, [BSA] = 0.315 mM, [BSA]:[**6**]:[**3**] = 0.125:1:400, 6 h, room temperature, Ar protection.

Supplementary Table 5. RAFT photopolymerization of **3** at the different light intensity.[a]

entry	Intensity (mW/cm^2)	M_n (kg/mol)	M_w/M_n	Conversion
1	0.8	92.5	1.18	53.7
2	1	100.9	1.15	63.04
3	1.9	103.1	1.14	79.38
4	3.6	105.9	1.16	85.99
5	4.3	116.3	1.19	91.8

[a] Polymerization conditions: [**3**] = 10% w/v, [BSA] = 0.315 mM, [BSA]:[**6**]:[**3**] = 0.125:1:400, 6 h, room temperature. Conversion ratio was determined by ^{1}H NMR spectrum in D_2O.

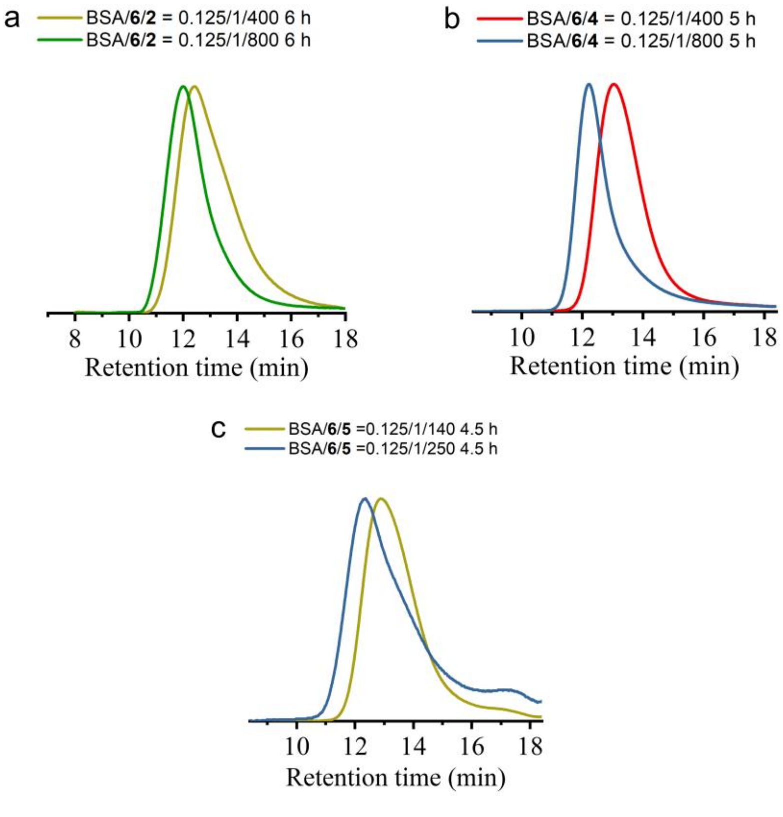

Supplementary Fig. 12. The GPC traces of BSA-mediated RAFT photopolymerization of (a) **2**, (b) **4** and (c) **5**, respectively. [BSA] = 0.315 mM, [**6**] = 1.07 mM, [M] = 10% w/v, violet LED light (20 W, 405 nm), room temperature, Ar protection.

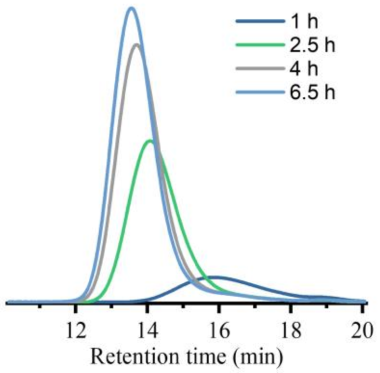

Supplementary Fig. 13. GPC traces of poly(**3**) in the ON/OFF experiments at different time intervals. [BSA] = 0.315 mM, [M] = 10% w/v, [BSA]:[**6**]:[**3**] = 0.144:1:460, violet LED light (20 W, 405 nm), room temperature, Ar protection.

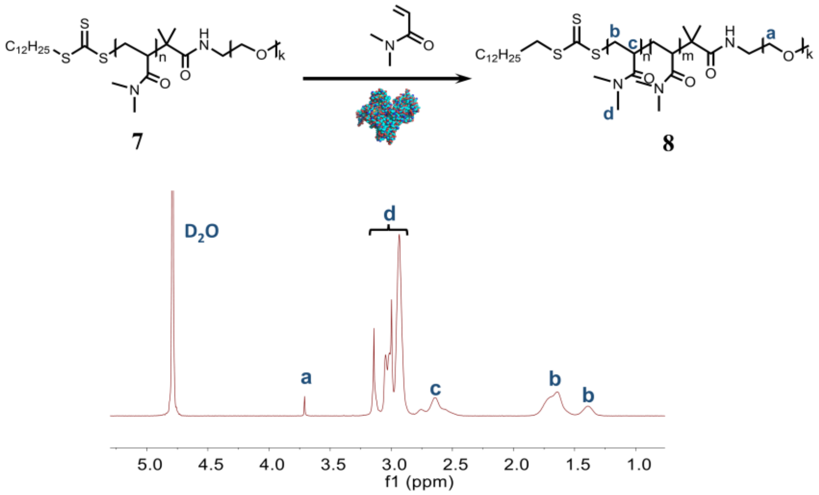

Supplementary Fig 14. ^{1}H NMR spectrum of the block copolymer **8** in D_2O. The first block polymer **7** was prepared using a molar ratio [BSA]:[**6**]:[**3**] = 0.162:1:517. The block copolymer **8** was prepared using a molar ratio [BSA]:[**7**]:[**3**] = 0.384:1:1230. violet LED light (20 W, 405 nm), room temperature, Ar protection.

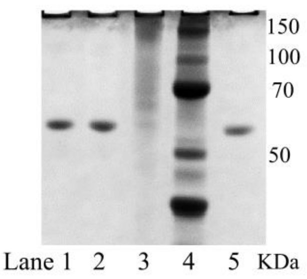

Supplementary Fig. 15. SDS-PAGE of BSA-mediated RAFT photopolymerization of **2**: Lane 1, solution before reaction; Lane 2, solution after 6 hours of reaction; Lane 3, BSA-poly(**2**) bioconjugates synthesized by literature[1] method; Lane 4, marker; Lane 5, mixture of BSA and **2**. The experiments were independently repeated three times with similar results.

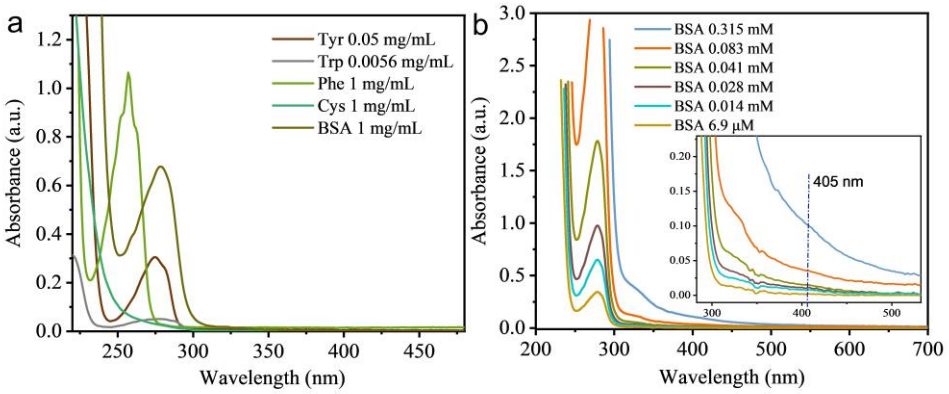

Supplementary Fig. 16. (a) UV-Vis absorption spectrum of BSA and various amino acids (L-phenylalanine, L-tryptophan, L-tyrosine, L-cysteine). Tyr which is mainly repeated units in BSA (20 per BSA) and much greater than tryptophan (2 per BSA), was mostly attributed to photo-oxidation sites of BSA. (b) The UV-Vis absorption spectrum of the different concentration of BSA.

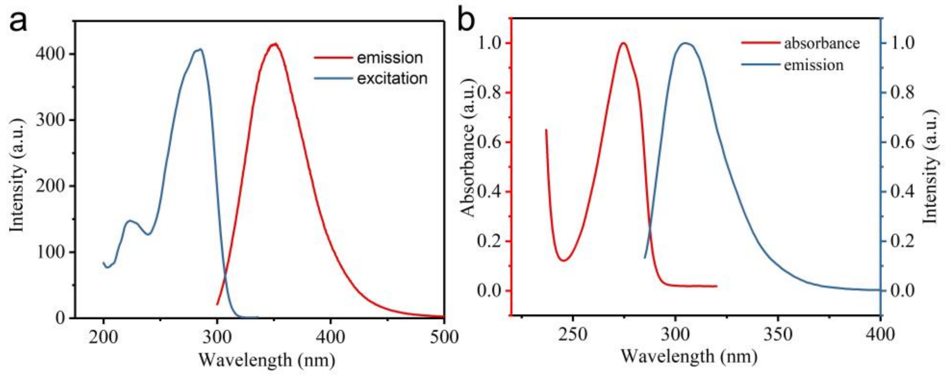

Supplementary Fig. 17. (a) Excitation and emission spectrum of BSA in H_2O. $\lambda_{max, ex} = 285$ nm, $\lambda_{max, em} = 350$ nm (ex = 280 nm). (b) Absorption spectrum and emission spectrum of Tyr. $\lambda_{max, abs} = 274$ nm, $\lambda_{max, em} = 304$ nm (ex=280 nm).

Supplementary Table 6. Excited-state oxidation potential of Tyr was estimated by the following equation:

$$E^*_{1/2} = E_{1/2} - \Delta E_{0,0} \quad (1)$$

$E^*_{1/2}$ is the excited-state redox potential; $E_{1/2}$ is the ground state redox potential; $\Delta E_{0,0}$ is the energy of the zero-zero transition of the fluorophore; The value of $\Delta E_{0,0}$ was calculated from the equation: $\Delta E_{0,0} = hc/\lambda$, where λ was estimated by the crossing point between the absorption spectrum and emission spectrum.

	$\triangle E_{0,0}$ (eV)	Eox (v) [2]	Eox* (v) [a]
Tyr	4.31	0.93	-3.38

[a] Eox*($Tyr^{\cdot+}$/Tyr*) = Eox($Tyr^{\cdot+}$/Tyr)-$E_{0,0}$(Tyr*/Tyr) = 0.93 - 4.31 = -3.38 V *vs* NHE.

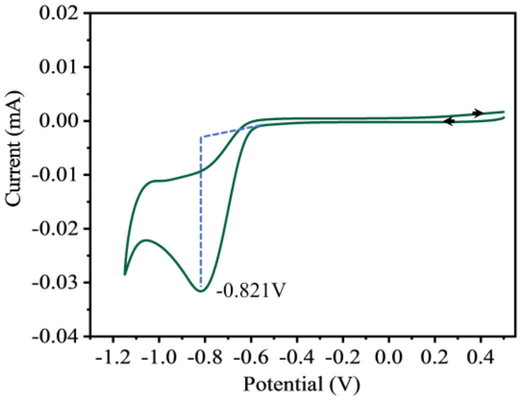

Supplementary Fig. 18. Cyclic voltammogram of **6** in 0.1 M KCl. Scan rate was 50 mV/s. The cathodic peak of **6** was observed at around -0.821 V (*vs* Ag/AgCl). Estimated reduction potential of **6** was -0.624 V (*vs* NHE).

Supplementary Table 7. The parameters of Stern-Volmer plots of BSA quenched by chain-transfer agent (**6**) or various monomers (**1**, **2**, **3**, **4**, EMA, IBMA), respectively.[a]

	**6**	**3**	**1**	**4**	**2**	EMA	IBMA
Kq*10^{-10} (L mol^{-1} s^{-1})	65.3967	0.971	0.23	1.4142	0.1499	0.1277	0.2885
R^2	0.9998	0.9993	0.9997	0.9978	0.9988	0.9896	0.9996

[a] Stern-Volmer relationship: $I_0/I = 1 + Kq \cdot \tau \cdot [Q]$. I_0 is the fluorescence intensity of chemical species without a quencher; I is the fluorescence intensity of chemical species with a quencher, Kq is the quencher rate coefficient; τ is the lifetime of the excited state of chemical species without a quencher; [Q] is the concentration of the quencher. R^2 is the correlation coefficient for the Stern-Volmer plots. $\tau = 10^{-8}$ s (BSA). EMA presents ethyl methacrylate. IBMA is isobutyl methacrylate.

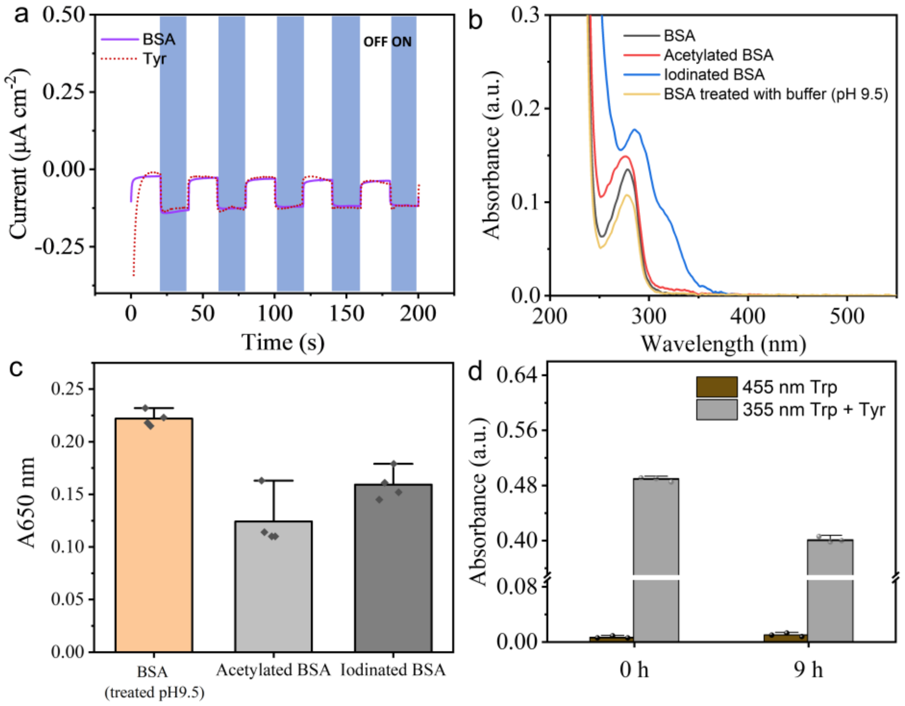

Supplementary Fig. 19. The study of the Tyr residues in BSA mediated polymerization. (a) The Photocurrent responses of BSA and Tyr with the 405 nm light switch. BSA and Tyr were immobilized by dropping 100 μL of 60 mg/mL BSA and 3.27 mg/mL Tyr suspension in 0.5% Nafion solution onto a FTO substrate surface, respectively, and evaporating the solvent at room temperature. Blocking experiments of the Tyr residues in BSA including acetylated BSA, iodinated BSA and BSA using pH 9.5 buffer to treat the same process used to mediate the photopolymerization of **1**[3,4]. (b) The UV-Vis absorption spectra of acetylated BSA, iodinated BSA and BSA treated pH 9.5 buffer. (c) The turbidity study of polymerization of **1** mediated by acetylated BSA, iodinated BSA and control BSA (treated pH 9.5 buffer) for 6 h, characterized by the visible light absorption intensity at 650 nm. According to previous reports, both “free” and “buried” tyrosyl residues of the Tyr residues in BSA were blocked by iodine. The “free” Tyr residues on BSA were blocked using N-Acetylimidazole. Data are presented as mean ± s.d., error bars indicate standard deviations (n = 4). (d) The determination of the amount of Tyr residues in BSA after polymerization for 9 h. The number of Tyr residues in BSA were determined by the method reported by J. CHRASTIL[5]. The number of Tyr was determined by UV-vis absorption at 355 nm. Data are presented as mean ± s.d., error bars indicate standard deviations (n = 3). [BSA] = 0.315 mM, [M] = 10% w/v, violet LED light (20 W, 405 nm), room temperature, Ar protection.

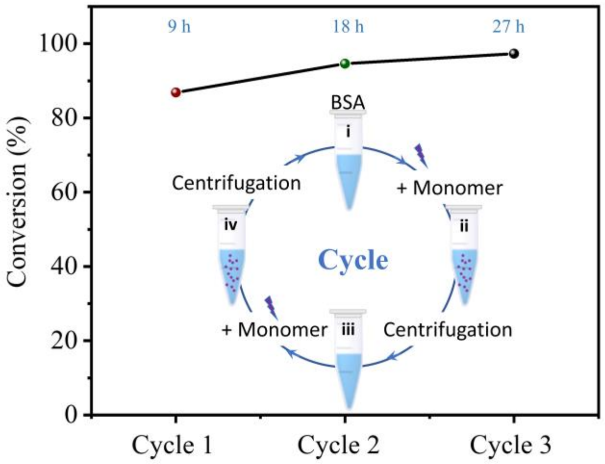

Supplementary Fig. 20. Plot of conversion ratio of photopolymerization of **1** mediated by recycled BSA. The inset was schematic illustration of BSA-mediated cycled photopolymerization of **1**.

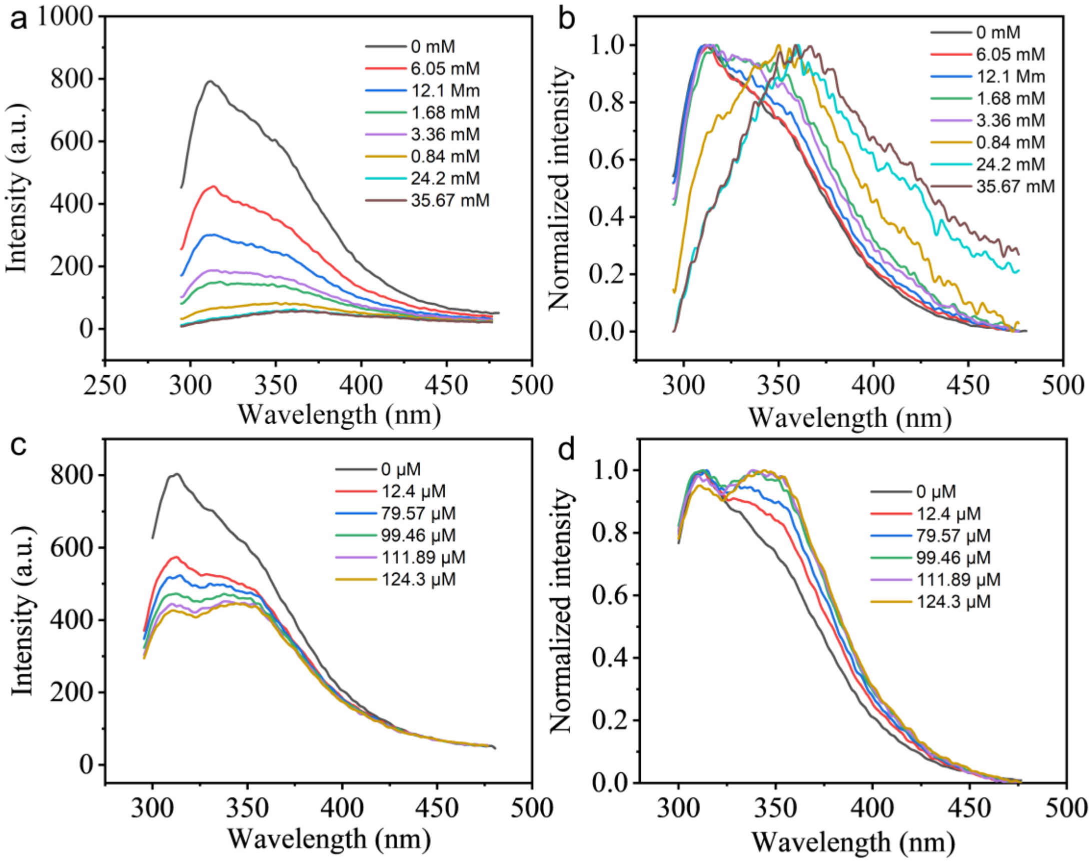

Supplementary Fig. 21. Fluorescence quenching emission spectrum of Ribonuclease A with the quenching agent of different concentrations, (a, b) **3** and (c, d) **6**. [RNase-A] = 0.128 mM.

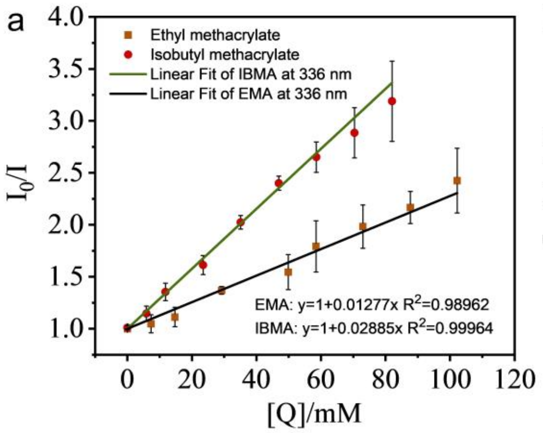

Supplementary Fig. 22. (a) Fluorescence quenching studies of BSA quenched with EMA and IBMA in H_2O. Stern-Volmer plots of the ratio I_0/I versus quencher concentration, EMA and IBMA. I_0 and I correspond to the emission intensity in the absence and presence of quencher, respectively. (b) Pseudo-first-order kinetic plot of Ln($[M]_0/[M_t]$) versus time of EMA and IBMA. Data are presented as mean ± s.d., error bars indicate standard deviations (n = 3).

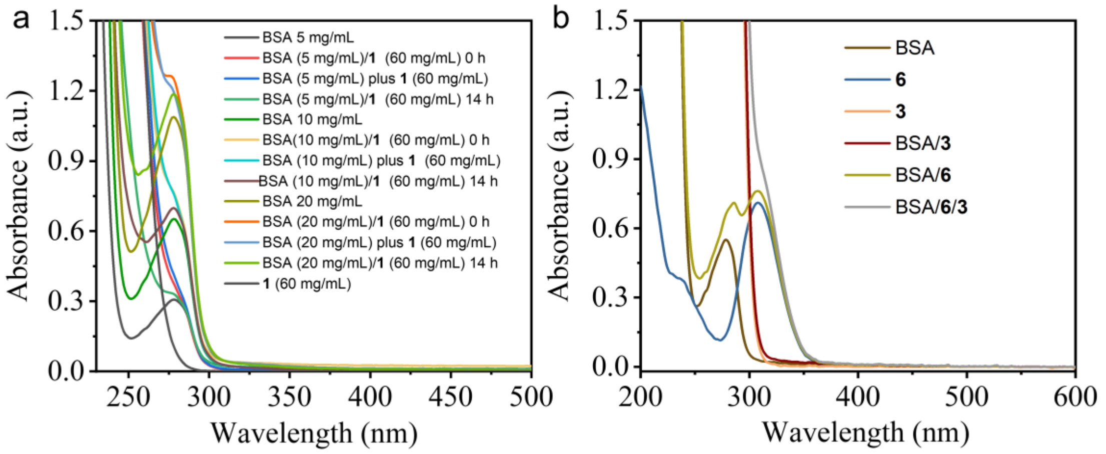

Supplementary Fig. 23. (a) The UV-Vis absorption spectrum of the mixture BSA/**1** solution before polymerization, the supernatant after polymerization for 14 h, separated BSA and **1**. (b) The UV-Vis absorption spectrum of BSA, **6** and **3**, either individually or in a mixture. [BSA] = 0.274 mM, [M] = 10% w/v, [**6**] = 1.163 mM.

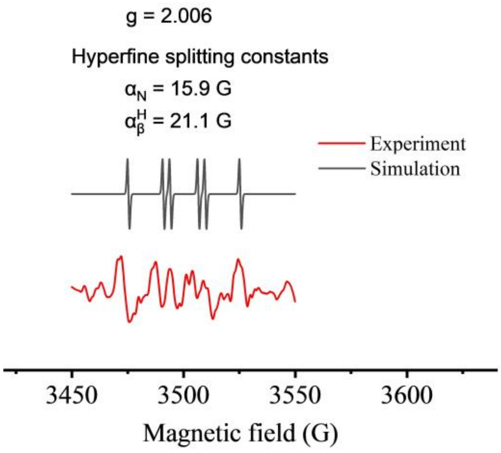

Supplementary Fig. 24. ESR spectrum of BSA mediated photopolymerization of **3**. [DMPO] = 100 mM, [**3**] = 1 M, [BSA] = 0.3 mM, violet LED light (20 W, 405 nm), room temperature, 3 h, Ar protection.

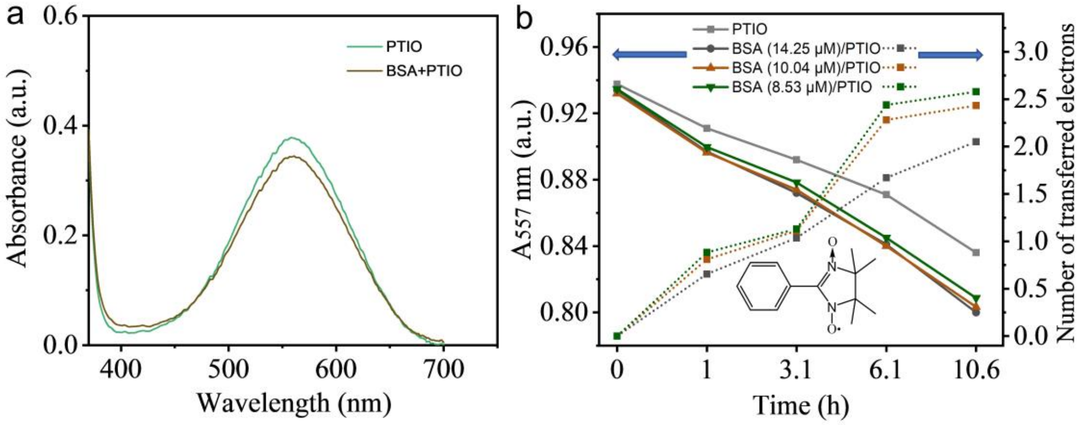

Supplementary Fig. 25. (a) The UV-Vis absorption spectrum of PTIO and the mixture PTIO/BSA under 405 nm LED light in H_2O. [PTIO] = 0.129 mM, [BSA] = 27 μM. (b) The UV-vis absorbance of PTIO at 557 nm and the estimated number of transferred electrons between BSA and PTIO versus time. The inset was the chemical structure of PTIO. We could estimate the number of electrons transferred between BSA and PTIO by monitoring the absorbance of PTIO at 557 nm. The number of transferred electrons was calculated by the equation: $N = C_{PTIO}(t\text{-}t_0)/C_{BSA}$, N is the number of electrons transferred between BSA and PTIO; $C_{PTIO}(t\text{-}t_0)$ is the consumption of PTIO calculated by variation of PTIO at 557 nm; C_{BSA} is the concentration of BSA. [PTIO] = [0.857 mM], Ar protection.

Supplementary Table 8. The parameters of proteins mediated RAFT photopolymerization.

entry	Protein	Mw (KDa)	Isoelectric point[a]	Tyr residues	Cofactor
1	BSA	66.4	5.6	20	-
2	HSA	66.4	5.79	18	-
3	Lysozyme	16.5	9.38	6	-
4	RNase-A	13.7	8.64	6	-
5	GOx	160	4.98	27	FAD

[a] Isoelectric point of proteins predicted by protein identification and analysis tools on the ExPASy Server[6]. All of the above cofactor-free proteins with the different molecular weight and isoelectric points can mediate the RAFT photopolymerization. Further, GOx containing the cofactor FAD, can also mediate the RAFT photopolymerization. Unlike previous literature report[7], the photopolymerization does not involve the catalytic process related to the cofactor, it was facilitated by Tyr residues in protein without the presence of substrate.

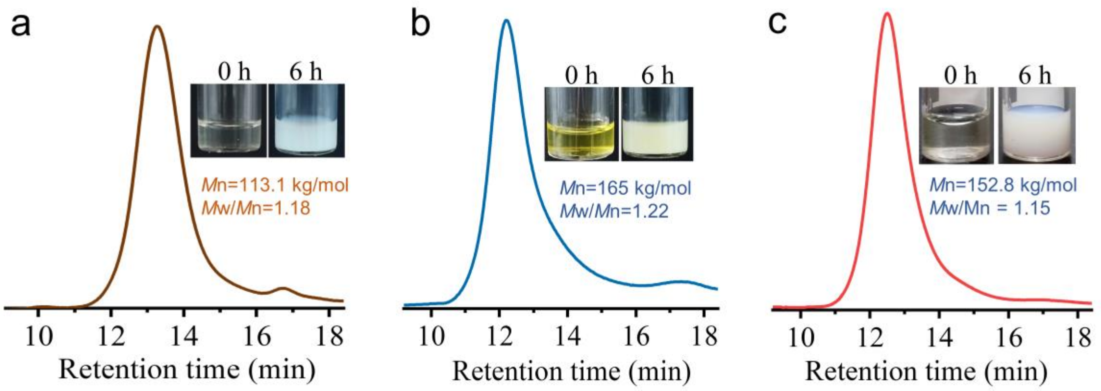

Supplementary Fig. 26. The GPC traces of RAFT photopolymerization of **1**, mediated by (a) GOx, (b) Lysozyme and (c) RNase-A, respectively. The insets were the photographs of polymerization at 0 h and 6 h. [GOx] = 0.048 mM, [Lysozyme] = 0.505 mM, [RNase-A] = 0.608 mM, [**6**] = 1.824 mM, [M] = 10% w/v, violet LED light (20 W, 405 nm), room temperature, Ar protection.

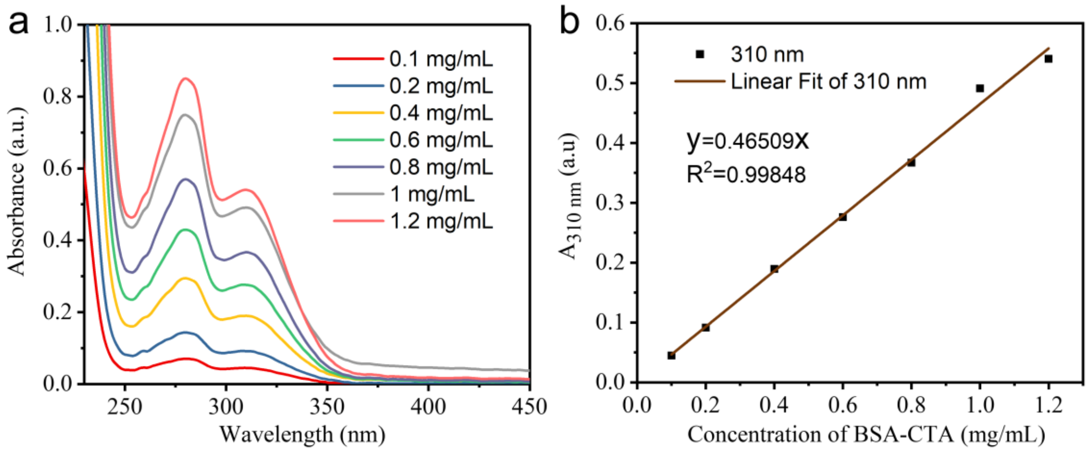

Supplementary Fig. 27. (a) UV-Vis absorption spectrum of macromolecular chain transfer agent BSA-CTA under different concentrations from 0.1 to 1.2 mg/mL, and (b) corresponding calibration curve based on plotting the absorbance at 310 nm against the concentrations of BSA-CTA.

Supplementary Table 9. The calculation of number of chain-transfer agent per BSA.

A310 nm	BSA-CTA (g/L)	CTA (μM)	BSA (μM)	nCTA:nBSA
0.04485	0.1	4.53055	1.48164	3.0578
0.09151	0.2	9.24394	2.96232	3.12051
0.18973	0.4	19.16569	5.92109	3.23685
0.27596	0.6	27.87627	8.88619	3.13703
0.36714	0.8	37.08688	11.84868	3.13004
0.49099	1	49.59766	14.79391	3.35257
0.54053	1.2	54.60198	17.77841	3.07125

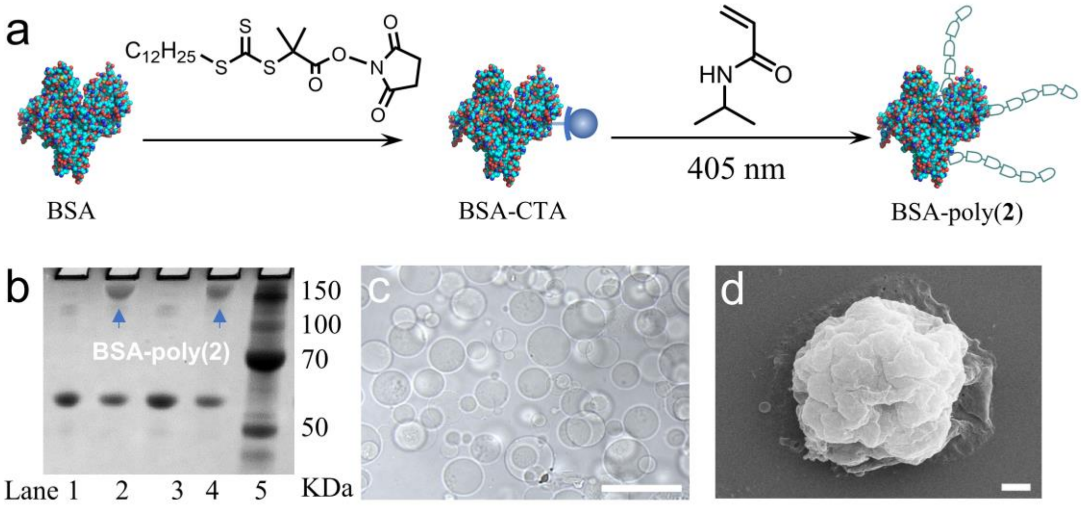

Supplementary Fig. 28. The utility of BSA-mediated RAFT photopolymerization of **2** to prepare BSA-poly(**2**) bioconjugates. (a) Schematic illustration of BSA-mediated RAFT photopolymerization of **2** to produce BSA-poly(**2**) bioconjugates. (b) SDS-PAGE of BSA-CTA-mediated RAFT photopolymerization of **2**: reaction solution before (Lane 1) and after polymerization for 9 hours (Lane 2) (molar ratio of BSA-CTA:**2** = 1:997); reaction solution before (Lane 3) and after polymerization for 9 hours (Lane 4) (molar ratio of BSA-CTA:**2** = 1:497); Lane 5, marker. The proteinsomes based on BSA-poly(**2**) obtained by method (a) were prepared by Pickering emulsion method. (c) Optical microscopy image of proteinsomes in water phase. Scale bar, 50 μm. The experiments were independently repeated three times with similar results in (b-c). (d) SEM images of proteinsomes in oil phase. Scale bars, 1 μm.

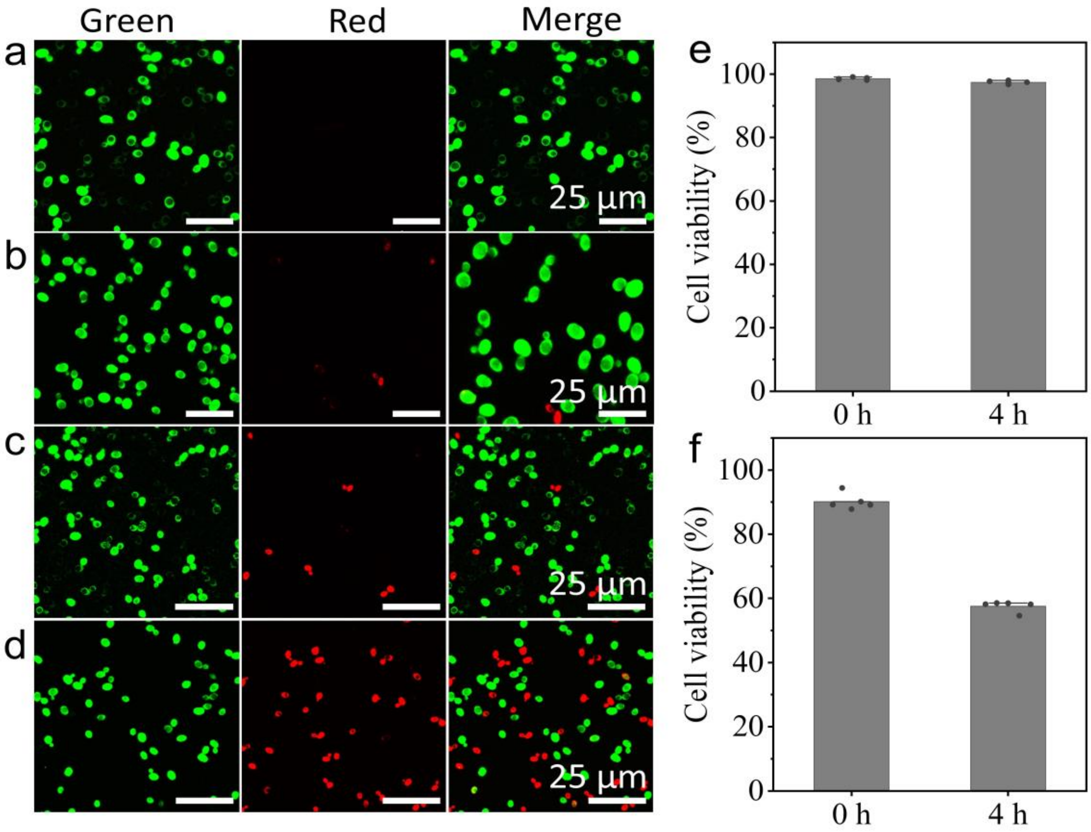

Supplementary Fig. 29. Confocal fluorescent microscopy images of yeast cells co-cultured with (a, b) 5%w/v **3** and (c, d) 10%w/v **3** under the light of 405 nm stained by FDA (green) and PI (red). Co-cultured for (a) 0 h; (b) 4 h; (c) 0 h; (d) 4 h, respectively. The experiments were independently repeated at least three times with similar results in (a-d). (e) The corresponding cell viability of a and b; (f) The corresponding cell viability of c and d. Data are presented as mean ± s.d., error bars indicate standard deviations (n = 5).

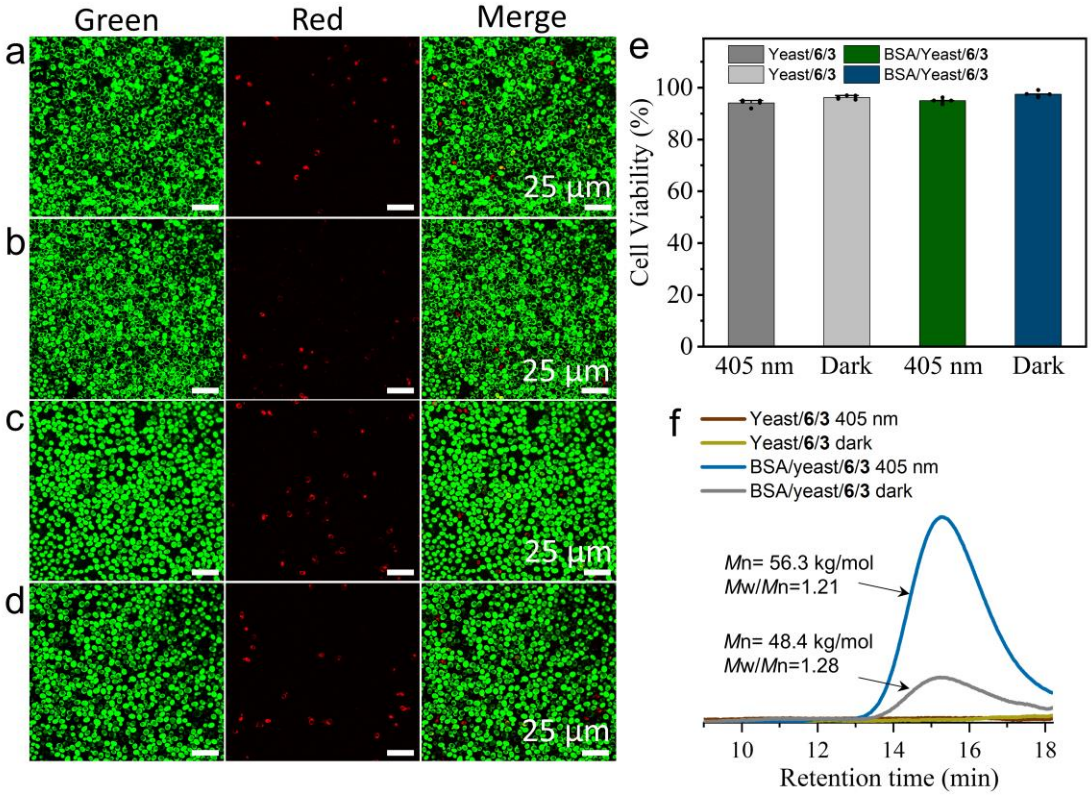

Supplementary Fig. 30. (a-d) Confocal fluorescent microscopy images of yeast cells after polymerization of **3** for 1.5 h in cells aqueous solution in the (a, b) presence or (c, d) absence of BSA, stained by FDA (green) and PI (red). (a) 405 nm; (b) dark; (c) 405 nm; (d) dark. The experiments were independently repeated at least three times with similar results in (a-d). (e) The cell viability after polymerization for 1.5 h. Data are presented as mean ± s.d., error bars indicate standard deviations (n = 5). (f) The GPC curves after polymerization for 1.5 h. [M] = 5% w/v, [BSA] = 0.15 mM.

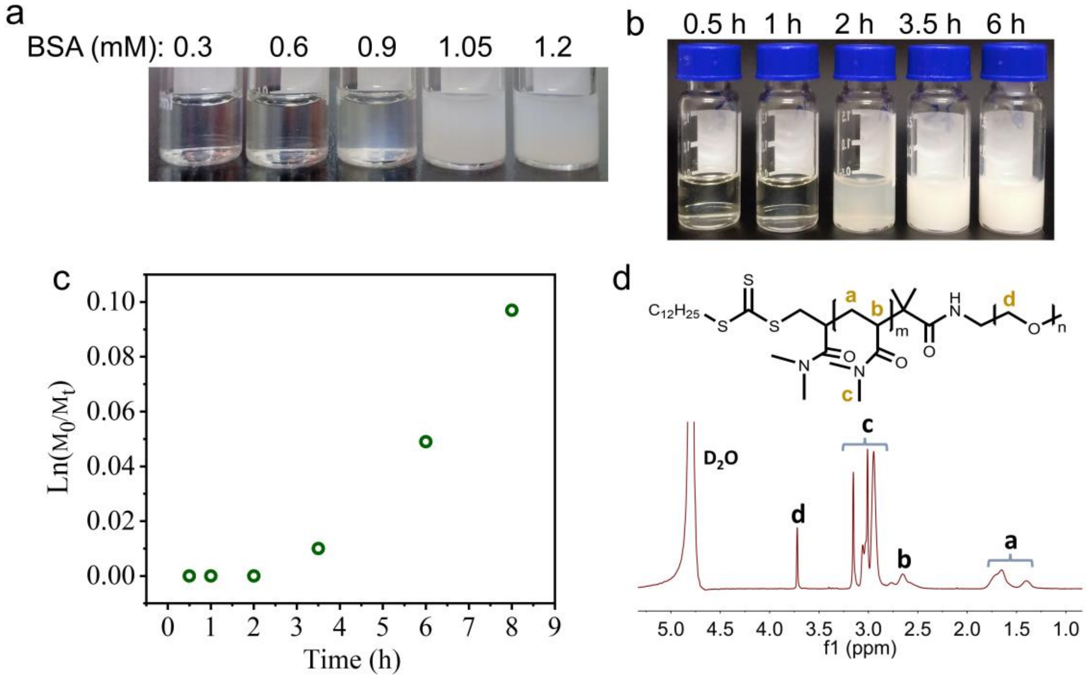

Supplementary Fig. 31. The investigation of aerobic photopolymerization. (a) Photograph of BSA-mediated photopolymerization of **1**. (b) Photograph of BSA-mediated photopolymerization of **1** versus time, and (c) the kinetic plot of Ln($[M]_0/[M_t]$) versus time of **3**, which revealed the induction periods of the polymerization at 1.5-2 h and the rate of oxygen consumption was estimated to be 0.155-0.117 μmol min^{-1}, [BSA] = 1.2 mM. (d) ^{1}H NMR spectrum of poly(**3**) in D_2O. [M] = 10% w/v, violet LED light (20 W, 405 nm), aerobic condition.

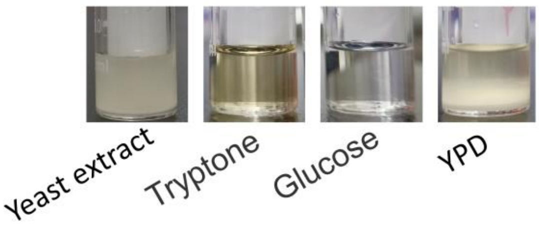

Supplementary Fig. 32. Photograph of YPD medium and its components-mediated photopolymerization of **1** at aerobic condition. Yeast extract (10 mg/mL), tryptone (20 mg/mL), glucose (20 mg/mL), [**1**] = 10% w/v, violet LED light (20 W, 405 nm), aerobic condition.

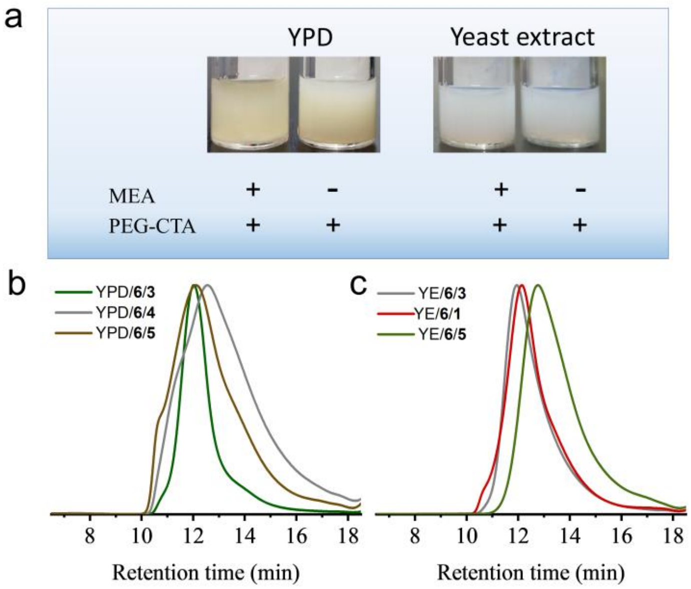

Supplementary Fig. 33. Aerobic photopolymerization was mediated by protein-containing biological media. (a) Photograph of photopolymerization of **1**, mediated by YPD medium and yeast extract (YE), respectively. The GPC traces of RAFT photopolymerization of the different monomers, mediated by protein-containing biological media, (b) YPD and (c) YE, respectively. [M] = 10% w/v, violet LED light (20 W, 405 nm), room temperature, aerobic condition.

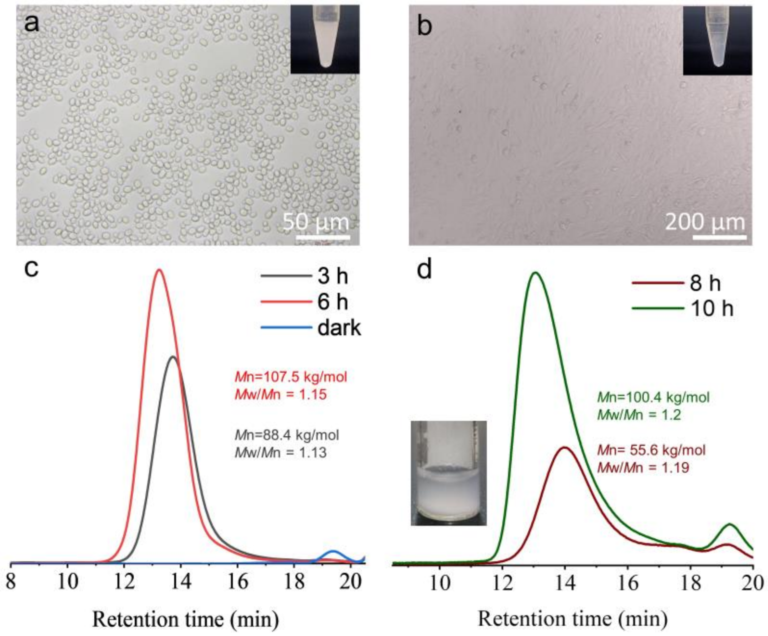

Supplementary Fig. 34. Cell lysates mediated photopolymerization. (a) Microscopy image of native yeast cells. The inset was photograph of yeast cells lysates. (b) Microscopy image of native NIH-3T3 cells (mouse-sourced stem cells). The inset was photograph of NIH-3T3 cells lysates. The experiments were independently repeated three times with similar results in (a-b). (c) GPC traces of RAFT photopolymerization of **3**, mediated by yeast cells lysates. (d) GPC traces of RAFT photopolymerization of **1**, mediated by NIH-3T3 cells lysates. The inset was photograph of photopolymerization of **1** for 10 h. Cells lysates were obtained by lyophilization and dispersed into H_2O. The synthesized poly(**1**) and poly(**3**) with narrow molecular weight distribution were observed. [Yeast cells lysates] = 5 mg/mL. NIH-3T3 cells cultured to the cell density shown in (b) in T25 cell culture flask were collected, and then lyophilization and dispersion in 1 mL of H_2O, [**6**] = 1.7 mM, [M] = 10% w/v, violet LED light (20 W, 405 nm), room temperature, Ar protection.

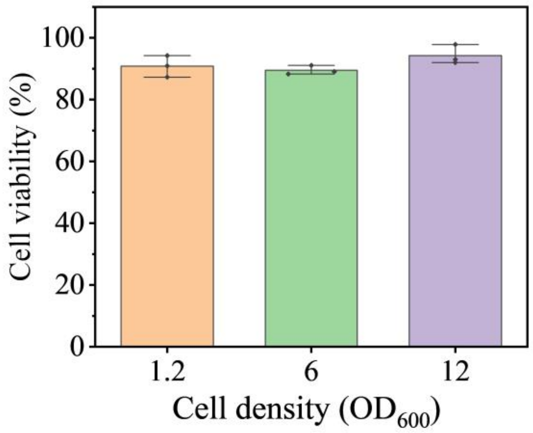

Supplementary Fig. 35. The cell viability statistics of yeast cells co-cultured with 2%w/v Nass for 24 h, stained by FDA (green) and PI (red). Data are presented as mean ± s.d., error bars indicate standard deviations (n = 3).

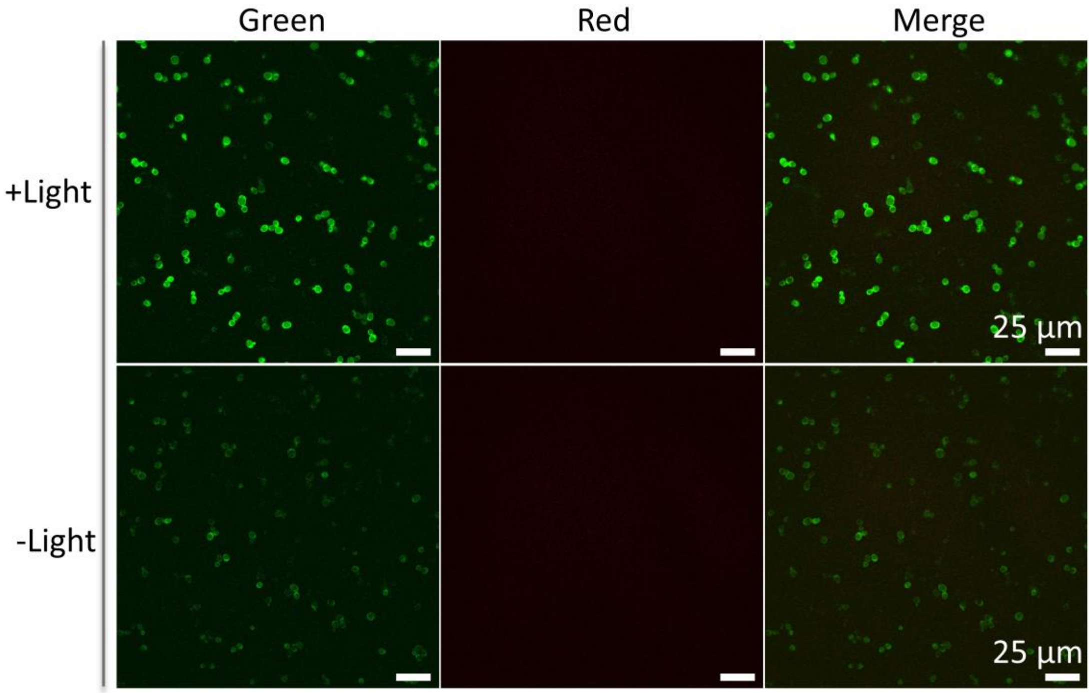

Supplementary Fig. 36. The confocal fluorescence microscopy images of yeast cells loaded with Nass and FMA (green) at 96 h after treated or untreated with 405 nm light for 40 min, stained by PI (red). The experiments were independently repeated three times with similar results.

Supplementary References

1. Huang, X. et al. Interfacial assembly of protein-polymer nano-conjugates into stimulus-responsive biomimetic protocells. *Nat. Commun.* **4**, 1-9 (2013).
2. Harriman, A. Further comments on the redox potentials of tryptophan and tyrosine. *Journal of physical chemistry*. **91**, 6102-6104 (1987).
3. Riordan, J. F., Wacker, W. E. & Vallee, B. L. N-Acetylimidazole: A reagent for determination of “free” tyrosyl residues of proteins. *Biochemistry-US*. **4**, 1758-1765 (1965).
4. Azari, P. R. & Feeney, R. E. The resistances of conalbumin and its iron complex to physical and chemical treatments. *Arch. Biochem. Biophys.* **92**, 44-52 (1961).
5. Chrastil, J. Spectrophotometric determination of tryptophan and tyrosine in peptides and proteins based on new color reactions. *Anal. Biochem.* **158**, 443-446 (1986).
6. Gasteiger, E. et a. Protein Identification and Analysis Tools on the ExPASy Server. (In) John M. Walker (ed): *The Proteomics Protocols Handbook*. 571-607 (Humana Press, 2005).
7. Zhou, F., Li, R., Wang, X., Du, S. & An, Z. Non-natural photoenzymatic controlled radical polymerization inspired by DNA photolyase. *Angew. Chem. Int. Ed*. **58**, 9479-9484 (2019).
