# Supplementary material for: Tyrosine residues initiated photopolymerization in living organisms
Source: Nat Commun. 2023 Jun 16;14:3598. doi: 10.1038/s41467-023-39286-8 (PMC10276049; doi:10.1038/s41467-023-39286-8)
Supplement: Supplementary file 3 — Reporting Summary [file 41467_2023_39286_MOESM3_ESM.pdf]

Corresponding author(s): Xin Huang, Xiaoman Liu

Last updated by author(s): Jun 2, 2023

Reporting Summary

Nature Portfolio wishes to improve the reproducibility of the work that we publish. This form provides structure for consistency and transparency in reporting. For further information on Nature Portfolio policies, see our Editorial Policies and the Editorial Policy Checklist.

Statistics

For all statistical analyses, confirm that the following items are present in the figure legend, table legend, main text, or Methods section.

n/a	Confirmed	
☐	☒	The exact sample size (n) for each experimental group/condition, given as a discrete number and unit of measurement
☐	☒	A statement on whether measurements were taken from distinct samples or whether the same sample was measured repeatedly
☐	☒	The statistical test(s) used AND whether they are one- or two-sided<br>*Only common tests should be described solely by name; describe more complex techniques in the Methods section.*
☒	☐	A description of all covariates tested
☐	☒	A description of any assumptions or corrections, such as tests of normality and adjustment for multiple comparisons
☐	☒	A full description of the statistical parameters including central tendency (e.g. means) or other basic estimates (e.g. regression coefficient) AND variation (e.g. standard deviation) or associated estimates of uncertainty (e.g. confidence intervals)
☐	☒	For null hypothesis testing, the test statistic (e.g. F, t, r) with confidence intervals, effect sizes, degrees of freedom and P value noted<br>*Give P values as exact values whenever suitable.*
☒	☐	For Bayesian analysis, information on the choice of priors and Markov chain Monte Carlo settings
☐	☒	For hierarchical and complex designs, identification of the appropriate level for tests and full reporting of outcomes
☒	☐	Estimates of effect sizes (e.g. Cohen's d, Pearson's r), indicating how they were calculated

Our web collection on statistics for biologists contains articles on many of the points above.

Software and code

Policy information about availability of computer code

Data collection	1H NMR spectra of monomer/polymer solutions were measured on a Bruker Avance-400 MHz Gel permeation chromatography (GPC) was performed on a Waters 1515 Isocratic HPLC pump, in an Intertek 81437 column oven operated at 40°C. Optical images were performed on a Leica DMi8 manual inverted fluorescence microscope at 40× magnifications. Confocal fluorescence images were performed on a Leica SP8 Laser scanning confocal microscope. Fluorescence spectra was performed by a fluorescence spectrophotometer (PerkinElmer, USA, LS 55). UV-vis spectra and turbidity measurements were measured on a PerkinElmer spectrophotometer (Lambda 750S, USA). Scanning electron microscopy (SEM) images of dried samples were obtained with a SU8000 instrument. Dynamic light scattering (DLS) measurements were performed using Malvern Zetasizer Nano-ZSP at 25°C using a scattering angle of 90°. Fourier transform infrared spectroscopy (FTIR) measurements were performed on PerkinElmer spectrometer with a LiTaO3 detector (Spectrum Two, USA). Cyclic voltammetric measurements were conducted on a CHI760E eletrochemical working station (Chenhua, Shanghai, China). Electron spin resonance (ESR) measurement was performed using Bruker EMX PLUS.
Data analysis	The statistics of the yeast cells and integrated optical density of gel was carried out using Image J Fiji. Data process was conducted using Origin 2021.

For manuscripts utilizing custom algorithms or software that are central to the research but not yet described in published literature, software must be made available to editors and reviewers. We strongly encourage code deposition in a community repository (e.g. GitHub). See the Nature Portfolio guidelines for submitting code & software for further information.

Data

Policy information about availability of data

All manuscripts must include a data availability statement. This statement should provide the following information, where applicable:

- Accession codes, unique identifiers, or web links for publicly available datasets
- A description of any restrictions on data availability
- For clinical datasets or third party data, please ensure that the statement adheres to our policy

All other experimental data supporting the findings of the study are available within the article and its Supplementary Information.

Human research participants

Policy information about studies involving human research participants and Sex and Gender in Research.

Reporting on sex and gender	Not applicable in this study.
Population characteristics	Not applicable in this study.
Recruitment	Not applicable in this study.
Ethics oversight	Not applicable in this study.

Note that full information on the approval of the study protocol must also be provided in the manuscript.

Field-specific reporting

Please select the one below that is the best fit for your research. If you are not sure, read the appropriate sections before making your selection.

☒ Life sciences ☐ Behavioural & social sciences ☐ Ecological, evolutionary & environmental sciences

For a reference copy of the document with all sections, see nature.com/documents/nr-reporting-summary-flat.pdf

Life sciences study design

All studies must disclose on these points even when the disclosure is negative.

Sample size	Dynamic light scattering (DLS) measurements were performed using Malvern Zetasizer Nano-ZSP at 25°C using a scattering angle of 90° to measure the diameter of poly(1) nanoparticles. Take 1 mL of poly(1) nanoparticles solution formed at the different polymerization time for testing (at least three replicates).
Data exclusions	No data were excluded from the analysis.
Replication	Each experiment was carried out at least three independent replications and was described in the figure legend with similar results.
Randomization	Yeast cells in each experiment were obtained by randomly selecting a single colony for expansion culture. Yeast cells were randomly grouped for experiments.
Blinding	The investigators were blinded to group allocation during data collection and/or analysis.

Reporting for specific materials, systems and methods

We require information from authors about some types of materials, experimental systems and methods used in many studies. Here, indicate whether each material, system or method listed is relevant to your study. If you are not sure if a list item applies to your research, read the appropriate section before selecting a response.

Materials & experimental systems

n/a	Involved in the study
☒	☐ Antibodies
☐	☒ Eukaryotic cell lines
☒	☐ Palaeontology and archaeology
☒	☐ Animals and other organisms
☒	☐ Clinical data
☒	☐ Dual use research of concern

Methods

n/a	Involved in the study
☒	☐ ChIP-seq
☒	☐ Flow cytometry
☒	☐ MRI-based neuroimaging

Eukaryotic cell lines

Policy information about cell lines and Sex and Gender in Research

Cell line source(s)	NIH-3T3 mouse-sourced stem cell sample was performed from ProCell, Shanghai, China.
Authentication	The NIH-3T3 cell line used has been anthenticated by STR profiling.
Mycoplasma contamination	The NIH-3T3 cell line used was not tested for mycoplasma contamination.
Commonly misidentified lines (See ICLAC register)	NIH-3T3 cell line used is not misdentified.
